# Identification of Pheromone Components of *Plagionotus detritus* (Coleoptera: Cerambycidae), and Attraction of Conspecifics, Competitors, and Natural Enemies to the Pheromone Blend

**DOI:** 10.3390/insects12100899

**Published:** 2021-10-02

**Authors:** Zoltán Imrei, Michael J. Domingue, Zsófia Lohonyai, Jardel A. Moreira, Éva Bálintné Csonka, József Fail, György Csóka, Lawrence M. Hanks, Miklós Tóth, Jocelyn G. Millar

**Affiliations:** 1 Plant Protection Institute, Agricultural Research Centre, ELKH, H-1022 Budapest, Hungary; lohonyai.zsofi@gmail.com (Z.L.); csonkaeva@yahoo.com (É.B.C.); toth.miklos@atk.hu (M.T.); 2 Department of Entomology, Kansas State University, 123 W. Waters Hall, Manhattan, KS 66506, USA; michael.j.domingue@usda.gov; 3 Department of Entomology, Institute of Plant Protection, Hungarian University of Agriculture and Life Sciences, H-1118 Budapest, Hungary; fail.jozsef@uni-mate.hu; 4 Brainfarma Industria Química e Farmacêutica S.A, Barueri 06400-000, Brazil; jardel.moreira@yahoo.com.br; 5 Department of Forest Protection, Forest Research Institute, University of Sopron, H-3232 Mátrafüred, Hungary; csoka.gyorgy@uni-sopron.hu; 6 Department of Entomology, University of Illinois at Urbana-Champaign, Urbana, IL 61801, USA; hanks@illinois.edu; 7 Department of Entomology, University of California, Riverside, CA 92521, USA; millar@ucr.edu

**Keywords:** headspace sampling, (*R*)-3-hydroxyhexan-2-one, (*S*)-2-hydroxyoctan-3-one, aggregation-sex pheromone, Clytini, predator, *Plagionotus detritus*, *Clerus mutillarius*, *Xylotrechus antilope*

## Abstract

**Simple Summary:**

The longhorn beetle, *Plagionotus detritus* (L.), occurs throughout Europe and into the Middle East. Our principal aim was to identify the male-produced aggregation-sex attractant pheromone of this species. A pheromone-based monitoring system could support plant protection in areas where it might become a pest and natural conservation in areas where it is endangered. Headspace volatiles were collected from live beetles and analyzed by coupled gas chromatography-electroantennogram detection and gas chromatography-mass spectrometry. Two components of the extracts that elicited responses from antennae of *P. detritus*, (*R*)-3-hydroxyhexan-2-one and (*S*)-2-hydroxyoctan-3-one were identified, (±)-3-hydroxyhexan-2-one was purchased, and (*S*)-2-hydroxyoctan-3-one was synthesized, and field-tested. The blend of the two components attracted both sexes of *P. detritus* in field bioassays. Unexpectedly, a predatory clerid beetle, *Clerus mutillarius* F., was also attracted by both of the synthetic compounds. Another longhorn beetle species, *Xylotrechus antilope* Schönh. was significantly attracted to traps baited with (*S*)-2-hydroxyoctan-3-one alone or the blend containing this compound. These results show that the pheromone components and combinations play key roles in longhorn beetle life history and ecology, and the community of species in which they live.

**Abstract:**

(1) Background: The principal aim of our work was to identify pheromone components for *Plagionotus detritus* (L.) (Coleoptera: Cerambycidae), which could be exploited for developing a pheromone-based monitoring system for the complementary purposes of plant protection in areas where it might become a pest, and natural conservation in areas where it is rare or endangered. (2) Methods: Collection and analysis of headspace volatiles were carried out with field-collected beetles. Bioactive volatile compounds identified [(*R*)-3-hydroxyhexan-2-one and (*S*)-2-hydroxyoctan-3-one] from extracts of males were purchased [(±)-3-hydroxyhexan-2-one], and synthesized [(*S*)-2-hydroxyoctan-3-one] and field-tested. Electroantennogram assays showed that antennae of the predatory beetle *Clerus mutillarius* F. (Coleoptera: Cleridae) also responded to the synthetic compounds. (3) Results: A two-component aggregation-sex pheromone consisting of (*R*)-3-hydroxyhexan-2-one and (*S*)-2-hydroxyoctan-3-one was identified for *P. detritus*. (±)-3-hydroxyhexan-2-one and (*S*)-2-hydroxyoctan-3-one attracted adults of *P. detritus* in field bioassays. Adults of the clerid *C. mutillarius* also were attracted to both compounds. The cerambycid *Xylotrechus antilope* Schönh was significantly attracted to traps baited with (*S*)-2-hydroxyoctan-3-one alone or the blend containing this compound. (4) Conclusions: Our data confirmed that 3-hydroxyhexan-2-one and 2-hydroxyoctan-3-one are male-produced pheromone components for *P. detritus*. These results show that both intraspecific and interspecific communication may play key roles in longhorn beetle life history and ecology, with closely and more distantly related species eavesdropping on each other’s signals.

## 1. Introduction

Saproxylic insects such as bark beetles and cerambycid beetles, which utilize live and dead woody plants as hosts, constitute crucial components of forest communities, beginning the process of recycling the nutrients from woody tissues back into the forest ecosystem. As such, they are sensitive to habitat loss through deforestation, or conversion of natural forest into intensively managed, often monocultural stands in which dead trees, fallen branches, and logging debris are rapidly removed, eliminating host materials for saproxylic species [1]. This scenario is evident in the patchwork of countries throughout Europe. Some insect species have suffered dramatic losses in terms of numbers and biodiversity in highly developed and densely populated countries with decreasing remnants of natural forests. Conversely, healthy and abundant populations of the same species may exist in countries with substantial amounts of relatively undisturbed woodlands and natural forests. Thus, monitoring the abundance and diversity of saproxylic species can constitute a useful indirect measure of forest health [2].

The longhorn beetle *Plagionotus detritus* (L.) (Coleoptera: Cerambycidae) occurs from Europe to Transcaucasia, North Iran, and Asia Minor [3,4]. This species has suffered widespread population decline from habitat loss, mainly due to modern forestry practices in Western Europe, and is now considered threatened and near extinction in several countries [5]. However, *P. detritus* remains a common saproxylic beetle in Central Europe, including Poland [6,7] and Hungary [3,8], in Southern Europe in Italy [9], Eastern Europe in Romania [3,10], Ukraine [11,12], and Russia [13]. The species has occasionally been designated as a pest because it can damage recently cut oak wood that is stored outdoors [14] and it is more abundant in semi-natural forests than in intensively managed forests [9]. Larvae develop in deciduous trees, especially in dead, dry (fallen or standing) trunks or thick branches of oaks (*Quercus* spp.), hornbeam (*Carpinus betulus* L.), beech (*Fagus sylvatica* L.), and sweet chestnut (*Castanea sativa* Mill.).

The principal aim of our work was to identify the key pheromone components involved in bringing the sexes of *P. detritus* together for mating. These pheromones could form the basis for developing a pheromone-based monitoring system for the complementary purposes of plant protection in areas where it might become a pest, and natural conservation in areas where it is endangered. When our experiments were started, the pheromone composition of *P. detritus* was not known. Thus, we began our studies with field observations of the diurnal activity patterns of *P. detritus* to determine the best times for collecting possible pheromonal volatiles from beetles of both sexes. We then analyzed the resulting extracts by coupled gas chromatography-electroantennogram detection (GC-EAD), focusing on sex-specific compounds which elicited antennal responses as putative pheromone candidates. These compounds were then identified by coupled gas chromatography-mass spectrometry (GC-MS), synthesized, and field-tested. In parallel, morphological studies were carried out to identify male-specific prothoracic gland pores as the likely sites of pheromone release.

During the course of our field trials, we found that the *P. detritus* pheromone blend also attracted a second cerambycid species, *Xylotrechus antilope* Schönh (Coleoptera: Cerambycidae), and a possible natural enemy, the predatory clerid beetle, *Clerus mutillarius* F. (Coleoptera: Cleridae), indicating that the pheromone components are perceived and exploited by other members of the forest community.

## 2. Materials and Methods

### 2.1. Sources of Insects

*Plagionotus detritus* beetles for collection of headspace volatiles were field-collected at Mátrafüred, Hungary (GPS coordinates: 47.841607N, 19.999709E) on or around recently cut, sunlit piles of sessile oak logs (*Quercus petraea* [Matt.] Liebl.) from 5 to 18 June 2012 and from 17 to 20 June 2013 (Online Resource 1, Table S1). The sex of the beetles was determined by the outer morphology of the abdominal tip [3]. The sexes were held separately in ventilated clear plastic boxes (56 × 28 × 28 cm) with freshly cut oak twigs, under outdoor temperature and light conditions.

During field bioassays with the possible pheromone components of *P. detritus*, significant numbers of the clerid beetle *Clerus mutillarius* were attracted to baited traps. Thus, beetles were collected at the same site under similar conditions around recently cut oak log piles on 1 and 2 July 2018, and beetles were kept under the conditions described for *P. detritus*.

### 2.2. Field Observations of Behaviour

While collecting beetles from 6 to 8 June 2012 from 8 a.m. to 6 p.m. as described above, we observed and recorded beetle activity during periods of both sunny and cloudy weather. Observations on beetle activities were conducted to identify the periods of possible pheromone production in their daily rhythm, so to time volatile collections.

### 2.3. Microscopy of External Morphology

To determine whether *P. detritus* males had sex-specific pheromone gland pores, insect prothoraces of both sexes were examined and photographed with a VHX-5000 digital microscope with a 500× objective lens (Keyence Co., Osaka, Japan) based on generalized findings among longhorn beetles by others [15].

### 2.4. Collection and Analysis of Insect-Produced Volatiles

Headspace volatiles were collected from adult *P. detritus* males and females (see Online Resource 1, Table S1) for detailed collection methods. Volatiles were trapped on activated charcoal, using a closed-loop stripping system with beetles held in glass jars. Adults of *P. detritus* generally were active from midmorning through early afternoon on sunny days (ZI, pers. obs.). Thus, headspace volatiles was collected during those hours. Volatiles were collected from a jar without beetles as system control.

Headspace extracts were analyzed at the Budapest laboratory with a 6890N gas chromatograph (Agilent Technologies Inc., Palo Alto, CA, USA), equipped with an HP-5 ms column (20 m × 0.32 mm × 0.25 μm film thickness; J&W Scientific, Folsom, CA, USA). Injections were made in split mode (injector temperature 220 °C), and the oven was programmed from 50 °C for 1 min, then increased at 5 °C/min rate to 230 °C, and was held for 10 min. The carrier gas was helium (flow rate: 4.0 mL/min, 56 cm/s, constant pressure: 112 kPa). Samples were also analyzed on an HP-5890 gas chromatograph (Hewlett-Packard, now Agilent) equipped with a DB-WAX column (30 m × 0.32 mm × 0.25-μm film thickness; J&W Scientific). Injections were made in split mode (injector temperature 215 °C), with an initial oven temperature of 31 °C for 1 min, then increased at 10 °C/min to 240 °C and held for 10 min. The carrier gas was helium (2.0 mL/min). Beetle-produced compounds were initially recognized by visual comparison of the chromatograms of headspace extracts from males and females to system controls using Agilent ChemStation software (version A.10.02). Male-specific compounds were tentatively identified by comparing their retention times with authentic standards on nonpolar (HP-5 ms) and polar (DB-WAX) columns. Extracts of volatiles were shipped to UC Riverside to confirm the identifications.

At UC Riverside, the extracts were reanalyzed with an Agilent 6890N gas chromatograph interfaced to a 5975C mass selective detector in EI mode (70 eV). The GC was fitted with an HP-5 column (30 m × 0.25 mm ID, Agilent), and the instrument was programmed from 40 °C for 1 min, then increased 10 °C/min to 280 °C, held for 20 min. Injections were made in splitless mode (250 °C). Compounds were identified by matching their mass spectra and retention times with those of authentic standards. Extracts were also analyzed on a chiral stationary phase Cyclodex B column (30 m × 0.32 mm ID, J&W Scientific) in conjunction with standards of racemic and enantiomerically enriched 3-hydroxyhexan-2-one (3-C6) and 2-hydroxyoctan-3-one (2-C8) to determine which enantiomers the insects produced. The column oven was programmed from 50 °C/1 min, 3 °C/min to 220 °C, with an injector temperature of 100 °C to minimize thermally-induced isomerization, injecting in split mode. The elution orders were (*R*)-3-C6 (13.30 min), (*S*)-3-C6 (14.46 min); (*R*)-2-C8 (22.02 min), (*S*)-2-C8 (22.30 min). Identifications were confirmed by coinjecting standards with aliquots of an insect extract.

Headspace extracts were also analyzed by coupled gas chromatography-electroantennogram detection (GC-EAD). The responses from antennae of male beetles were recorded with glass electrodes (1.17 mm i.d., Ockenfels Syntech GmbH, Kirchzarten, Germany) pulled to a fine point and filled with 0.1 M KCl solution. These were attached to silver/silver chloride electrodes held in MP15 micromanipulators and connected to an IDAC 232 amplifier (Syntech). Extracts of volatiles were injected into a 6890N gas chromatograph (Agilent), equipped with the above-mentioned HP-5 column, and analyzed with a temperature program as described above. The column effluent was split between two identical pieces of deactivated fused silica capillary column (100 cm × 0.25 mm ID) connected to a four-way splitter. The fourth arm provided additional carrier gas. One capillary led to the flame ionization detector (FID 280 °C) and the other to the heated EAD port (220 °C; Gerstel ODP2 transfer line). The EAD capillary effluent was delivered to the antennal preparation in a stream of charcoal-filtered and humidified air in a glass tube (8 mm ID × 150 mm; airflow 500 mL/min).

### 2.5. Electroantennography (EAG) with Clerid Beetle Antennae

Electroantennography was used to test whether antennae of *P. detritus* of either sex responded to extracts of volatiles collected from male or female beetles. It was also used to test whether *C. mutillarius* males and females perceived the pheromone compounds identified in the headspace volatiles from *P. detritus* males. *Clerus mutillarius* beetles were sexed by dissection of the genitalia after the antennae had been removed. For EAG assays, an antenna from a live female (*n* = 6) or male (*n* = 7) adult *C. mutillarius* was excised at the base and mounted between two glass capillary electrodes containing 0.1 M KCl solution. A constant humidified airflow of ~0.7 L/min was directed over the antenna, placed ~3 mm from the exiting airflow from a Teflon™-coated steel stimulus delivery tube. One of the electrodes was grounded, and the other was connected to a high-impedance DC amplifier (IDAC-232; Ockenfels Syntech GmbH). Test compounds (100 ng) dissolved in 10 µL isopropyl alcohol were administered to a 5 × 5 mm piece of filter paper inside a Pasteur pipette. Stimuli were delivered by puffing 1 mL of air through the Pasteur pipette into the air stream flowing over the antenna. Isopropyl alcohol was used as the solvent control, while pure air was the blank control without any chemical stimulus. Response amplitudes were normalized against the means of responses to the standard (*Z*)-3-hexenol, which was tested before and after the test compounds.

### 2.6. Sources of Chemicals

Racemic 3-C6 (CAS number 54123-75-0) was purchased from Bedoukian Research, Inc. (Danbury, CT, USA), 2,3-hexanedione from Aldrich Chemical Co. (Milwaukee, WI, USA), and an analytical sample of 2,3-octanedione was obtained by oxidation of 2-hydroxyoctan-3-one with pyridinium dichromate in methylene chloride [16]. A small amount of (*R*)-3-C6 was available from a previous synthesis [17], in which 94% enantioenriched material was prepared by enzymatic kinetic resolution of the racemic material, which was sufficient for analytical samples but not enough for field trials. The (*S*)-enantiomer of 2-C8 was prepared by modification of the route described by Hall et al. (2006) for the synthesis of the homologous enantiomers of 2-hydroxydecan-3-one, as described below.

*Preparation of (S)-ethyl 2-(2-tetrahydropyranloxy)-propanoate (**2**).* 3,4-Dihydro-2*H*-pyran (53.40 g, 635 mmol) was added dropwise over 30 min to a stirred solution of (*S*)-ethyl lactate **1** (50.0 g, 423 mmol) and 100 mg p-toluenesulphonic acid in 120 mL CH_2_Cl_2_ at 0 °C, under Ar. The mixture was warmed to room temperature and stirred until all the starting material had been consumed (~5 h). The mixture was then diluted with diethyl ether (60 mL) and washed with saturated aqueous NaHCO_3_ and brine, dried, and concentrated. The crude product was Kugelrohr distilled (64–68 °C, 0.25 mm Hg), yielding 81.4 g of protected alcohol **2** (95%) as a mixture of diastereoisomers (2:1, measured by GC). ^1^H NMR (major stereoisomer): δ 1.26 (t, *J* = 7.2 Hz, 3H), 1.44 (d, *J* = 7.0 Hz, 3H), 1.48–1.90 (m, 6H), 3.47–3.55 (m, 1H), 3.80–3.88 (m, 1H), 4.11–4.25 (m, 3H, 2 quadruplets overlapped with signals of other stereoisomer), 4.66–4.72 (m, 1H, dd overlapped with signals of other stereoisomer). ^1^H NMR (minor stereoisomer): δ 1.27 (t, *J* = 7.2 Hz, 3H), 1.38 (d, *J* = 6.8 Hz, 3H), 1.48–1.90 (m, 6H), 3.40–3.47 (m, 1H), 3.88–3.95 (m, 1H), 4.11–4.25 (m, 2H-quadruplet overlapped with signals of other stereoisomer), 4.40 (q, *J* = 7.0 Hz, 1H), 4.66–4.72 (m, 1H, dd overlapped with signals of other stereoisomer). MS (rel. intensity) *m/z*: 144 (1), 130 (3), 129 (4), 101 (19), 85 (100), 73 (11), 67 (12), 57 (16), 55 (17), 45 (25), 43 (24).

*Preparation of lithium (S)-2-(2-tetrahydropyranloxy)-propanoate (**S-3**)*. Ethyl-(*S*)-2-(2-tetrahydropyranloxy)-propanoate **2** (80.0 g, 396 mmol) was added to a stirred suspension of LiOH^.^H_2_O (16.60 g, 396 mmol) in ethanol (200 mL) under Ar at 0 °C. The reaction was stirred for 30 min at 0 °C, then warmed to room temperature and stirred for 3 h. The mixture was then concentrated under vacuum, and hexane (50 mL) was added to the concentrate followed by concentration under vacuum again to remove traces of ethanol and water by azeotroping. This procedure was repeated four times and then the residue was pumped under vacuum (0.1 mm Hg) for 10 h. ^1^H NMR showed that ethanol was still present in the salt, and so the crude lithium lactate salt (26.0 g) was suspended in benzene (100 mL), the benzene was distilled off at atmospheric pressure to azeotrope off ethanol and water traces, and the residue was pumped under vacuum again for 10 h, affording pure lithium (*S*)-2-(2-tetrahydropyranloxy)-propanoate ***S*-3** as a light yellow solid. ^1^H NMR (major stereoisomer): δ 1.39 (d, *J* = 6.8 Hz, 3H), 1.42–1.92 (m, 6H), 3.40–3.55 (m, 1H), 3.82–3.92 (m, 1H), 4.18 (q, *J* = 6.8 Hz, 1H), 4.70 (t, *J* 3.5 Hz, 1H). ^1^H NMR (minor stereoisomer): δ 1.34 (d, *J* = 6.8 Hz, 3H), 1.42–1.92 (m, 6H), 3.40–3.55 (m, 1H), 4.00–4.05 (m, 1H), 4.12 (q, *J* = 7.0 Hz, 1H), 4.54 (dd, *J* = 2.3 and 7.0 Hz, 1H). Some multiplets from the two diastereomers overlapped.

*Preparation of 2(S)-(2-tetrahydropyranloxy)-octan-3-one (**S-4**)*. Lithium wire (1% sodium content; 1.39 g, 200 mmol, previously rinsed with hexane) was cut into small pieces directly into a three-necked flask charged with anhydrous diethyl ether (85 mL) under Ar. A few drops of a solution of 1-bromopentane (15.1 g, 100 mmol) in 15 mL ether were added and the mixture was stirred at ambient temperature until the reaction started. The mixture was then cooled to −20 °C and the remaining solution of bromopentane was added dropwise over 1.5 h. When the addition was complete, the mixture was allowed to warm to room temperature and stirred for an additional hour before using. The resulting solution of pentyllithium was then added dropwise to a suspension of lithium (*S*)-2-(2-tetrahydropyranloxy)-propanoate ***S*-3** (11.26 g, 62.5 mmol) in Et_2_O (140 mL) under Ar at −30 °C. The reaction was allowed to warm to room temperature, stirred overnight, then poured into ice and extracted with Et_2_O (3 × 100 mL). The combined organic layers were washed with saturated aqueous NH_4_Cl solution and brine, dried over Na_2_SO_4_, and concentrated. The residue was purified by vacuum flash chromatography on silica gel (hexane: EtOAc, 9:1), affording 2(*S*)-(2-tetrahydropyranloxy)-octan-3-one ***S*-4** (12.60 g) in 88.3% yield. ^1^H NMR δ (major stereoisomer) 0.87 (t, *J* = 7.2 Hz, 3H), 1.20–1.33 (m, 4H), 1.35 (d, *J* = 7.0 Hz, 3H), 1.47–1.92 (m, 8H), 2.41 (dt, *J* = 7.4 and 17.4 Hz, 1H), 2.51 (dt, *J* = 7.4 and 17.4 Hz, 1H), 3.45–3.52 (m, 1H), 3.83–3.90 (m, 1H), 4.27 (q, *J* = 7.0 Hz, 1H), 4.54 (dd, *J* = 3.1 and 4.7 Hz, 1H). (Some signals are overlapped with signals of the other stereoisomer). MS (major stereoisomer) (rel. intensity) *m/z*: 184 (1), 129 (3), 101 (1), 99 (2), 85 (100), 71 (4), 67 (10), 57 (13), 55 (9), 43 (25), 41 (18). ^1^H NMR δ (minor stereoisomer) 0.87 (t, *J* = 7.2 Hz, 3H), 1.20–1.33 (m, 4H), 1.25 (d, *J* = 6.8 Hz, 3H), 1.47–1.92 (m, 8H), 2.57 (dt, *J* = 7.4 and 17.7 Hz, 1H), 2.64 (dt, *J* = 7.4 and 17.7 Hz, 1H), 3.40–3.45 (m, 1H), 3.77–3.83 (m, 1H), 4.07 (q, *J* = 6.8 Hz, 1H), 4.60 (dd, *J* = 2.6 and 5.1 Hz, 1H). (Some signals are overlapped with signals of other stereoisomer). MS (minor stereoisomer) (rel. intensity) *m/z*: 184 (1), 129 (2), 101 (1), 99 (2), 85 (100), 71 (4), 67 (10), 57 (14), 55 (9), 43 (25), 41 (18). 

*Preparation of (S)-2-hydroxyoctan-3-one (**S-5**).* Pyridinium *p*-toluenesulfonate (55 mg) was added to a stirred solution of 2(*S*)-(2-tetrahydropyranloxy)-octan-3-one ***S*-4** (12.5 g, 54.8 mmol) in methanol (80 mL) at 0 °C, under Ar. The reaction was allowed to warm to rt and stirred overnight. Methanol was removed under reduced pressure and water (40 mL) was added to the residue, and the mixture was extracted with Et_2_O (4 × 30 mL). The organic phase was washed with saturated NaHCO_3_ solution and brine, dried over Na_2_SO_4_ and concentrated. Purification by vacuum flash chromatography on silica gel (Hexane/EtOAc, 95:5 as eluent) followed by Kugelrohr distillation (bp ~50–52 °C at 0.05 mm Hg) gave (*S*)-2-hydroxyoctan-3-one ***S***-**5** (5.48 g, 69.4%, ee 98.1%, Cyclodex B GC column, see conditions above). ^1^H NMR δ 0.88 (t, *J* = 7.0 Hz, 3H), 1.20–2.34 (m, 4H), 1.36 (d, *J* = 7.1 Hz, 3H), 1.57–1.66 (m, 2H), 2.41 (dt, *J* = 17.0 and 7.4 Hz, 1H), 2.50 (dt, *J* = 17.0 and 7.4 Hz, 1H), 3.57 (d, *J* = 3.8 Hz, 1H), 4.20 (dq, *J* = 3.7 and 7.2 Hz, 1H). ^13^C NMR δ 14.09, 20.06, 22.61, 23.50, 31.59, 37.70, 72.78, 212.95 ppm. MS (rel. intensity) *m/z*: 144 (M^+^, 1), 116 (1), 111 (1), 101 (31), 100 (16), 99 (91), 88 (9), 83 (67), 74 (9), 71 (53), 57 (12), 55 (86), 45 (66), 43 (100), 41 (31).

### 2.7. Field Test 1

*Pheromone lures.* For the first bioassay, pheromone release devices consisted of press-sealed polyethylene bags (50 × 75 mm, 50 µm wall thickness, #018161A, Fisher Scientific, Pittsburgh, PA, USA), attached with a metal clip to the traps (without puncturing the bags). Traps were baited with lures containing 50 µL of racemic 3-C6 and (*S*)-2-C8 individually and in 1:1 binary blends, diluted in 0.5 mL isopropanol for single components or 1 mL for the blend of components. A dental cotton roll (Celluron^®^, Paul Hartmann AG, Heidenheim, Germany) was placed in the bags to act as a reservoir and minimize leakage. Lures were loaded with pheromone solutions immediately before being deployed. Pheromone solutions were prepared in advance and stored at −18 °C until needed.

*Traps*. Field tests were carried out with funnel traps (Figure S1) consisting of a funnel trap body emptying into a collection bucket and surmounted with a transparent upper funnel, a vertical plastic sheet that enlarges the trap funnel to intercept flying insects. The version with a light green funnel sheet has been shown to be highly effective in catching *Plagionotus floralis* (Pallas), a congener of *P. detritus* [18,19] and with a transparent funnel sheet in catching *Molorchus umbellatarum* Schreb [20] and *Plagionotus arcuatus* L. (Coleoptera: Cerambycidae) [21]. To reduce the potentially confounding effects of the light green visual stimulus, a transparent funnel was used in the test of the pheromone compounds. The inner surface of the funnel was coated with a Teflon^®^ emulsion (95% polytetrafluoroethylene-based spray; B’laster Corporation, Cleveland, OH, USA) to increase trapping efficiency [22,23], and a Vaportape^®^ insecticidal strip (Hercon Environmental Inc., Emigsville, PA, USA) was placed in the collection bucket. The pheromone dispenser was suspended from the vertical plastic sheet to hang in the middle of the funnel opening.

*Experimental site.* The experiment was conducted in a sessile oak forest at Mátrafüred (GPS coordinates: 47.841559N, 19.999626E), Hungary between 5 June–23 July 2013 (Test 1). Traps were set up in a randomized complete block design, with 4 blocks per site. Traps were spaced 10 m apart, and within 10 m of oak log piles (*Q. petraea*), where adults of *P. detritus, P. arcuatus*, *X. antilope*, and *C. mutillarius* were abundant. Traps were mounted at ground level on metal posts. Traps were inspected once weekly, when captured insects were collected. Lures were replaced every second week. Captured beetles were identified as species using the key of Kaszab [3]. Voucher specimens have been deposited at the Hungarian laboratory.

### 2.8. Field Test 2

In field test 2, the same Teflon^®^-coated funnel traps were used but with two different colored upper funnels; transparent and light green. The transparent upper funnel was used as a control when the visual effect of the light green funnel sheet was tested in traps without pheromone lures. Field test 2 was conducted at the same site as field test 1, between 5 June–23 July 2014, with the same design and methods.

### 2.9. Statistics

Statistical analysis of trap catch data was conducted in R (R Core Team, 2017) and figures were produced using the software packages “dplyr” [24] and “ggplot2” [25]. Because even transformed trap catch data did not meet the assumptions of parametric tests, the non-parametric Kruskal–Wallis test was used [26]. When the Kruskal–Wallis test indicated significant differences, pairwise comparisons were made with Wilcoxon tests [27]. EAG data were analysed with ANOVA, with means separated by Fisher’s Protected LSD test (StatView® v4.01 and Super ANOVA® v1.11; Abacus Concepts Inc., Berkeley, CA, USA).

## 3. Results

### 3.1. Diurnal Activity of Beetles

*Plagionotus detritus* adults were active in the field from midmorning until the early afternoon, with beetles being most active during sunny periods. The beetles crawled or flew actively in sunny patches but remained relatively inactive in shaded areas, or during cloudy weather, even at high temperatures (25–35 °C). These observations were taken into account when setting up collections of headspace volatiles from live beetles in the laboratory.

### 3.2. Sex-Specific Pheromone Gland Pores

**Figure 1:**
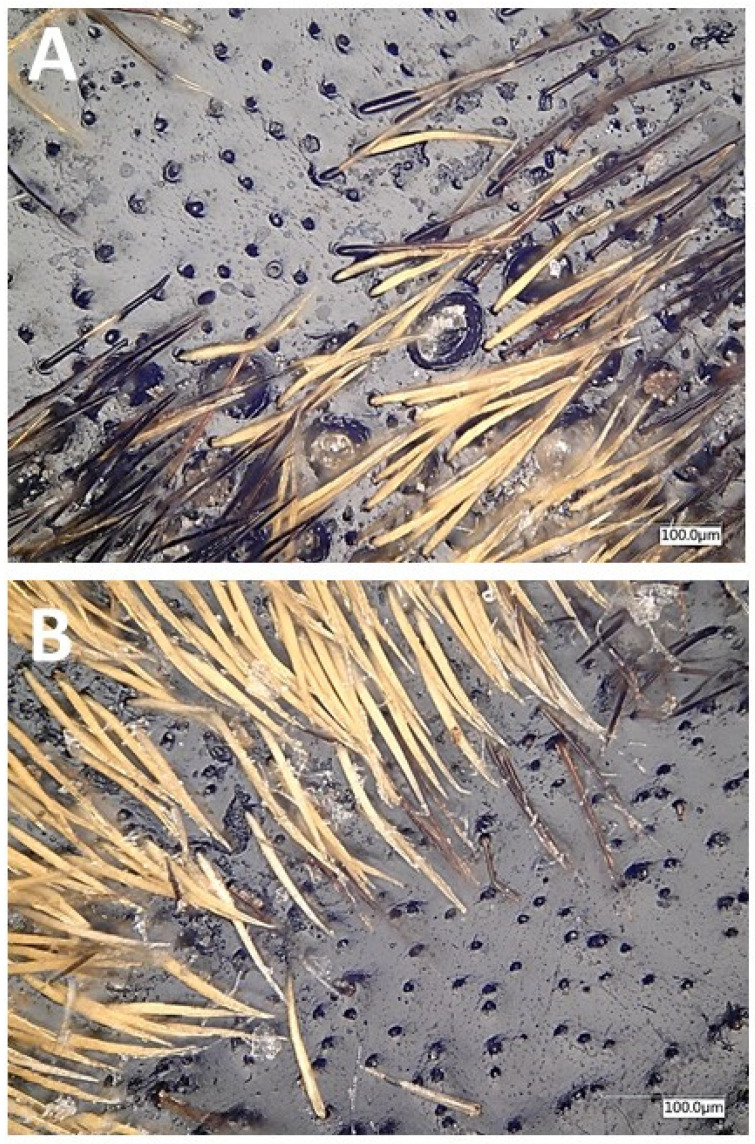
Dorsal surface of the prothoraces of male (**A**) and female (**B**) *Plagionotus detritus*. The oval-shaped pores found in males were absent in females (magnification 500×).

Similar to many other species in the subfamily Cerambycinae [15,28], we found sex-specific pores on the prothoraces of male *P. detritus*, which were not observed on the prothoraces of females (Figure 1). The prothoracic pores were oval, with a perceivable depth under the magnification used.

### 3.3. Collection and Analysis of Insect-Produced Volatiles

**Figure 2:**
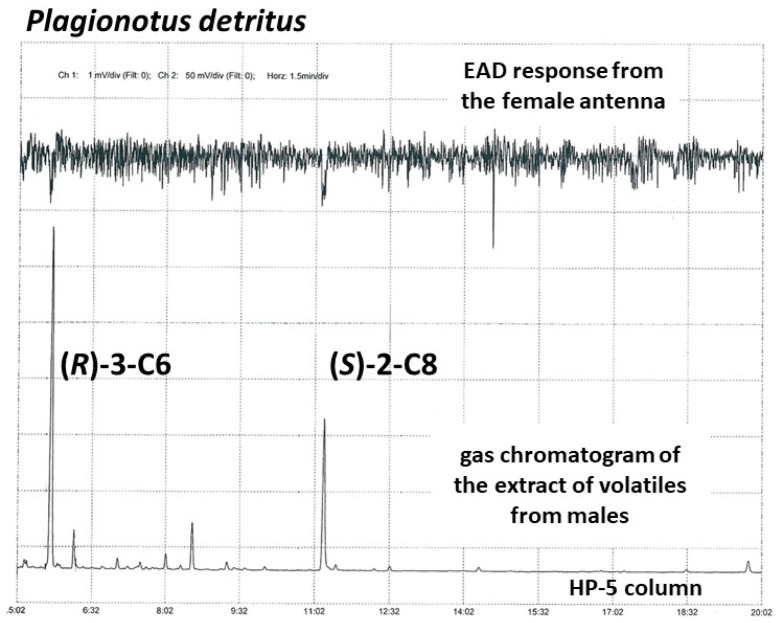
Representative GC-EAD trace of antennal response profile of a female *Plagionotus detritus* to male-produced volatiles. Top trace shows the antennal response, the bottom trace shows the corresponding gas chromatogram. Antennal responses to (*R*)-3-hydroxyhexan-2-one [(*R*)-3-C6] and (*S*)-2-hydroxyoctan-3-one [(*S*)-2-C8] are indicated.

**Figure 3:**
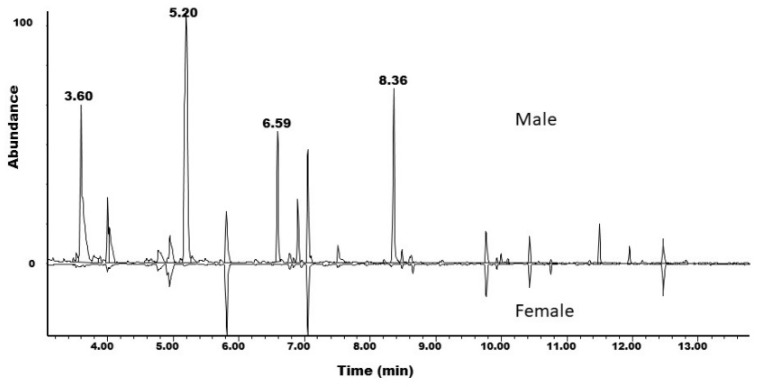
Total ion chromatograms of extracts of volatiles from male (top) and female (bottom, inverted) *Plagionotus detritus*. Compound identifications: retention time 3.60 min, 2,3-hexanedione; 5.20 min, (*R*)-3-hydroxyhexan-2-one; 6.59 min, 2,3-octanedione; 8.36 min, (*S*)-2-hydroxyoctan-3-one.

Preliminary EAG tests indicated that antennae from both sexes of *P. detritus* responded to volatiles collected from male beetles, while analogous volatiles from females did not elicit electrophysiological responses. Coupled GC-EAD analyses of the extracts of males showed two peaks which elicited consistent responses from antennae of both sexes (Figure 2). Subsequent analyses of the extracts of male *P. detritus* beetles by GC-MS resulted in the identifications of the compounds as 3-hydroxyhexan-2-one and 2-hydroxyoctan-3-one, based on comparisons of their mass spectra and retention times with those of authentic standards. 2,3-Hexanedione and 2,3-octanedione, degradation products of the two hydroxyketones, were also present in significant amounts (Figure 3). The compounds were present in an average ratio of 46% 3-C6:2-C8 (*n* = 4), but the ratios were highly variable, ranging from 10% to 78% 3-C6. Neither 3-C6 nor 2-C8 were present in analogous extracts from females. Further analyses of the extracts of males on a chiral stationary phase GC column revealed that the beetles produced (*R*)-3-hydroxyhexan-2-one and (*S*)-2-hydroxyoctan-3-one.

### 3.4. Field Tests

**Figure 4:**
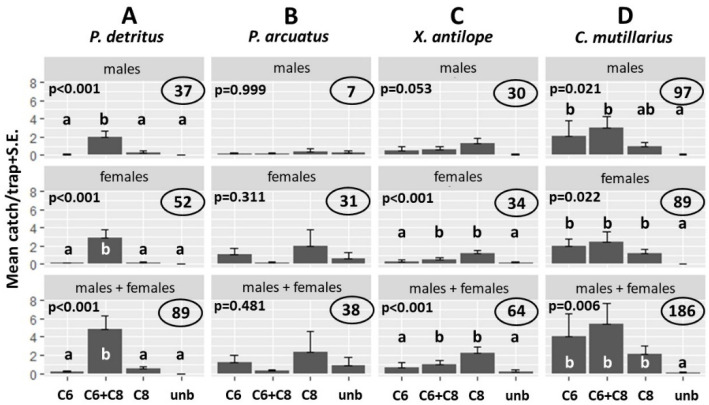
Means (+S.E.) of *Plagionotus detritus* (**A**), *Plagionotus arcuatus* (**B**), *Xylotrechus antilope* (**C**) and *Clerus mutillarius* (**D**) male and female specimens captured in traps baited with racemic 3-hydroxyhexan-2-one (C6), (*S*)-2-hydroxyoctan-3-one (C8), the combination of the two compounds, or unbaited controls. Columns with the same letter within a diagram are not significantly different at *P* = 0.05 by Kruskal–Wallis, followed by Wilcoxon non-parametric tests. *P* values in the upper left corner is a diagram show Kruskal–Wallis results if not significant at *P* = 0.05. Numbers in ovals in the right upper corner of individual diagrams give a total number of beetles captured.

*Field test 1.* Both males and females of *P. detritus* were significantly attracted to traps baited with the 1:1 blend of racemic 3-C6 and (*S*)-2-C8, and the sexes were caught in similar numbers (Figure 4A, Table S2). In contrast, traps baited with the individual compounds were not attractive, with catches of *P. detritus* in traps baited with the unique compounds being not significantly different than the unbaited controls.

*Plagionotus arcuatus* was also caught during the field bioassays. However, captures showed no clear tendencies across the different treatments (Figure 4B), with no significant differences among any of the treatments.

*Xylotrechus antilope* was significantly attracted to traps baited with (*S*)-2-C8 alone or the blend of racemic 3-C6 and (*S*)-2-C8 (Figure 4C), whereas traps baited with racemic 3-C6 alone were not significantly different than controls. Traps baited with the combination of the two volatiles did not differ from traps baited with (*S*)-2-C8 alone for females, or both sexes combined.

Unexpectedly, significant numbers of the clerid beetle *C. mutillarius* were attracted to the test lures. Thus, traps baited with racemic 3-C6 alone and the binary blend attracted significant numbers of males, while all three pheromone treatments attracted significant numbers of females, and males and females combined (Figure 4D).

*Field test 2.* Unbaited light green traps that attract and catch the congener *P. floralis* were not attractive to *P. detritus* over the seven-week flight period of this species, when the beetles were abundant. During this period, only four specimens of an atypical species of the habitat, *P. floralis* were caught in the light green traps, with zero catches in the transparent traps. Furthermore, neither of these traps showed any signs of being inherently attractive to *P. arcuatus*, *C. mutillarius,* or *X. antilope*, which were abundant during the seven weeks this field trial was deployed.

**Figure 5:**
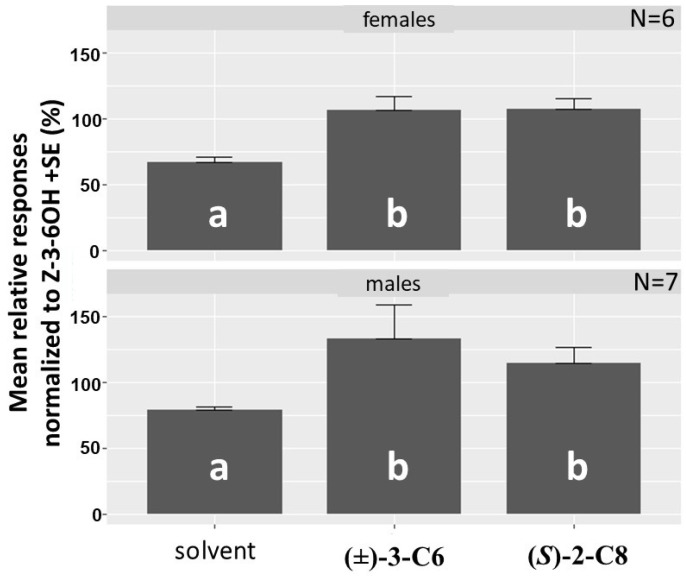
Normalized electroantennogram (EAG) responses of *Clerus mutillarius* to 100 ng doses of racemic 3-hydroxyhexan-2-one [(*±*)-3-C6] and (*S*)-2-hydroxyoctan-3-one [(*S*)-2-C8] expressed as means of seven males and six females. Means followed by the same letter are not significantly different, ANOVA and Fisher’s Protected LSD test (*P* < 0.05). Responses were normalized to the response of (*Z*)-3-hexenol (= 100%).

*Clerus mutillarius EAG.* One hundred nanogram doses of both racemic 3-C6 and (*S*)-2-C8 elicited significantly greater responses from antennae of both male and female *C. mutillarius* than the solvent control when puffed over the beetle antennae (Figure 5), indicating that the antennae were detecting both of these compounds from male *P. detritus*.

## 4. Discussion

Several longhorn beetle species in the tribe Clytini, including the study species *P. detritus*, *P. arcuatus,* and *X. antilope,* live in sympatry in the oak forests of the Mátra mountains of Hungary based on our observations over the past decade. Their flight periods also overlap, and aggregations of the beetles can be observed on sunlit boles and log piles [3]. The predatory clerid beetle *C. mutillarius* also occurs in large numbers, where it preys on bark beetles and other insect species [29].

As suggested by previous studies with other cerambycids in the subfamily Cerambycinae [15,28], the presence of sex-specific pores on the prothoraces of male *P. detritus* (Figure 1) indicated that these are likely to be where the male-specific pheromone components are produced and released. Analogous pores had been previously noted in the congener *P. arcuatus* [15,21], for which male-produced aggregation-sex pheromones have been recently identified [21].

Analyses of extracts of volatiles from both sexes of *P. detritus* by GC-EAD and GC-MS suggested that (*R*)-3-C6 and (*S*)-2-C8 are components of the male-produced pheromone of this species, and these results were corroborated by field trials. The blend of the two compounds attracted both males and females, indicating that (*R*)-3-C6 and (*S*)-2-C8 are aggregation-sex pheromone components, analogous to those found in many other species in the subfamily Cerambycinae [30]. Using a Central European (Hungary) population of *P. detritus,* our work confirms the results of Molander et al. [5], who characterized the same two compounds from a Swedish population. In Sweden, the ratio of the 3-C6 and 2-C8 compounds ranged between 100:16 and 100:52 (3-C6:2-C8). In contrast, in the present study, the ratio found in a composite sample from several extracts was 100:47 (3-C6:2-C8), indicating that the two geographically separated populations nevertheless produced similar ratios. However, it must be mentioned that the blend ratio has not been optimized for either population, and so testing a range of ratios of the two components could lead to the more effective attraction of beetles.

Whereas both the 5:1 ratio of 3-C6:2-C8 used by Molander et al. [5] and the 1:1 ratio used in the present study were significantly attractive, the individual compounds were not attractive to either population of *P. detritus*. Furthermore, the effects of other possible attractive stimuli, such as host volatiles or visual cues, have not yet been investigated. Field observations of aggregations of beetles on log piles of their hosts suggest that such cues could also be exploited to increase the attraction of beetles to traps.

There have been several recent reports of *P. detritus* being attracted to “generic” cerambycid lures, consisting of blends of a number of known pheromone compounds. For example, in field trials, adults of *P. detritus* were attracted to lures containing one of its pheromone compounds, 3-C6, and an isomer of the second pheromone component, 3-hydroxyoctan-2-one [7,9,31]. In the isomer, the hydroxyl and carbonyl functional groups are reversed as compared to 2-C8. This might suggest that both the 6- and 8-carbon hydroxyketones are necessary for *P. detritus* attraction, but the relative position of the hydroxyl- and carbonyl-functional groups (i.e., 2,3 vs. 3,2) may be less critical [31]. It is also possible that under field conditions, the 3-hydroxyoctan-2-one isomerizes to the 2-hydroxyoctan-3-one, because it is well known that these alpha-hydroxyketone motifs are readily interconverted, e.g., [32]. Alternatively, intraguild eavesdropping that enables *P. detritus* to perceive pheromone compounds of other longhorn beetle species may also explain the unexpected catches achieved by the regioisomer of 3-hydroxyoctan-2-one.

*Plagionotus arcuatus*, which is a common saproxylic beetle in most parts of Europe [8,33,34,35,36], was also trapped during the field bioassays. Its primary hosts include various species of oak (*Quercus* spp.), with larvae developing in the wood and under the bark of branches and trunks of recently dead or cut trees [37]. *Plagionotus arcuatus* occasionally is considered a pest because it can damage fresh oak wood stored outdoors [14,38]. However, *P. arcuatus* catches showed no clear tendencies (Figure 4B), although racemic 3-C6 was tested, which includes (*R*)-3-hydroxyhexan-2-one, the major pheromone component of *P. arcuatus* [21]. Our results are congruent with Swedish and Hungarian field tests [21], which showed that the 3-C6 component alone was insufficient to attract this species and that blends of this compound with the minor components 3-hydroxyoctan-2-one and 3-hydroxydecan-2-one are required in order to achieve attraction. This is consistent with the lack of attraction seen in the present study.

Another longhorn beetle in the subfamily Cerambycinae, *X. antilope*, was also caught in significant numbers during this study. This species occurs from Europe to Transcaucasia, North Iran, Asia Minor, and North Africa [3]. Typically, it is found at 400–800 m above sea level within Hungary. Larvae develop exclusively in thick branches of oaks and typically occur in dead wood and cut logs [3]. *Xylotrechus antilope* is considered rare and is red-listed by the International Union for Conservation of Nature (IUCN) among endangered species. Its populations are severely fragmented due to logging and wood harvesting in parts of northern Europe [39,40].

In the present study, *X. antilope* was attracted by 2-C8 as a single component, confirming earlier results [40], where the *S*-enantiomer of 2-C8 was identified as the major and likely only component of its pheromone. Males of several longhorn beetle species, particularly in the genus *Xylotrechus*, produce 2-C8, which is also produced by male *Plagionotus christophi* [41,42].

*Xylotrechus antilope* has also been reported to be attracted to mixtures of known pheromones of other cerambycine species, such as 3-hydroxyhexan-2-one, 3-hydroxyoctan-2-one, and *syn*-2,3-hexanediol combined with ethanol [9,31]. However, in tests with 3-hydroxyoctan-2-one as a single component, with the position of the hydroxyl and carbonyl groups interchanged from its known pheromone component 2-hydroxyoctan-3-one, no attraction was observed [31]. Thus, its attraction to blends of pheromones of heterospecifics may be due to eavesdropping on the communication of other guild members rather than a widening of the tuning of its own pheromone system. Exploiting the pheromones of heterospecifics, combined with cues from stressed hosts (e.g., ethanol) to locate suitable larval hosts, may help in finding relatively scarce host resources, such as fallen trees.

Adults of the predatory clerid beetle *C. mutillarius* are common on the ground and bark of old deciduous trees in mostly oak and beech forests [43,44] in Central and Southern Europe, and North Africa [45,46]. *Clerus mutillarius* is considered a bark beetle predator [47], but no experimental data is available in the literature on its range of prey.

In the present study, both racemic 3-C6 and (*S*)-2-C8 attracted adults of *C. mutillarius.* Furthermore, EAG assays demonstrated that antennae from both sexes could perceive these compounds in biologically relevant amounts. These data suggest that the common cerambycid pheromone components racemic 3-C6 and (*S*)-2-C8 might function as kairomones for *C. mutillarius,* which help this species to locate prey directly or indirectly through attracting the clerids to habitats where prey, including eggs and larvae of cerambycids, might be found. The latter possibility seems more likely because during the course of our fieldwork, despite *P. detritus* and *C. mutillarius* beetles being observed on the same logs in high abundance, we did not observe any instances of the clerids attacking the adult cerambycids. It has been reported that *C. mutillarus* larvae feed on bark beetle larvae, and that adult *C. mutillarius* feed on bark beetle adults [48]. Another clerid, *Opilo domesticus* Sturm (Coleoptera: Cleridae), is known to prey on larvae of the longhorn beetle *Hylotrupes bajulus* L. (Coleoptera: Cerambycidae) [48].

There have been previous reports of clerid beetles being attracted by cerambycid semiochemicals. For example, in North America, traps baited with ethanol and *syn*-2,3-hexanediol attracted *Chariessa pilosa* (Forster) (Cleridae) independently from the presence of ethanol [49]. This species may be exploiting the signals from cerambycids to either locate prey, or to locate suitable larval habitats. Overall, it is not uncommon for clerids to exploit pheromone components of their prey as kairomones. For example, *Thanasimus formicarius* (L.) (Coleoptera: Cleridae) responds to racemic ipsdienol and ipsenol and to a lesser extent to (*S*)-*cis*-verbenol [50]. All three of these compounds are pheromone components of several bark beetles in the genus *Ips*, on which the clerid preys.

*Further optimization of trapping methods to achieve a more effective monitoring tool.* There are several possible ways that the effectiveness of pheromone-based trapping of *P. detritus* might be improved, including optimizing the lure blend, incorporating host plant cues into the lures, and testing different trap colours and heights. For example, it was recently demonstrated that both *P. detritus* and *X. antilope* were more effectively trapped at heights of 4–15 m as compared to 1.5 m above ground [9]. Our field bioassays were carried out at ground level in a sunlit, open area near host logs, where both longhorn beetle species could be observed in large numbers on sunny days. We took advantage of this by placing our traps close to the sunlit log piles where beetles were aggregating, and where the freshly cut logs were releasing host tree volatiles. However, it is often a challenge to find log piles of the respective host trees, because they are quickly removed to sawmills because of their high economic value.

In published studies, purple Lindgren 12-funnel traps were found to be somewhat better for catching *P. detritus* than green traps [9,51]. As a non-flower visitor, *P. detritus* may use an achromatic visual channel, which could explain its attraction to a dark color such as purple. According to Rassati et al. [9], *Clerus mutillarius* was caught significantly more in red and brown traps than in black traps, but black traps caught more individuals than yellow, green, and grey traps [46]. In our second field test during the present study, we saw no evidence that any of our study species were attracted to unbaited light green traps, which had previously been shown to attract *P. floralis* [19], a known flower feeder. The lack of response to these traps by *P. detritus*, *P. arcuatus*, and *X. antilope* suggests that these species may not use flowers as a primary food source. However, *X. antilope* is strongly attracted by yellow traps even if it is not a flower-visiting species, which might be linked to mate finding due to the yellow stripes present on the beetle elytra [51].

The three cerambycid species described in this study are sympatric and have similar flight seasons, creating the potential for cross attraction given that they share some pheromone components. However, upon closer examination, some mechanisms for preventing cross attraction are suggested. For example, *P. detritus* requires both 3-C6 and 2-C8 to be attracted, and so would not be cross-attracted by 2-C8 alone, the pheromone of *X. antilope*, or the three-component blend of *P. arcuatus*, which lacks 2-C8. Similarly, *P. arcuatus* requires a three-component combination consisting of 3-C6 with the homologs 3-hydroxyoctan-2-one and 3-hydroxydecan-2-one to be attracted effectively would not be attracted by either of the other two species. Thirdly, *X. antilope* would not be attracted by *P. arcuatus*, which does not produce 2-C8, and Figure 4C suggests that *X. antilope* may be inhibited by 3-C6, a component of the *P. detritus* pheromone.

## 5. Conclusions

In summary, our data confirmed that (*R*)-3-C6 and (*S*)-2-C8 are male-produced pheromone components for *P. detritus*, and that (*S*)-2-C8 is a pheromone component for *X. antilope*. Adults of the predatory clerid beetle *C. mutillarius* were attracted to both compounds. These results suggest that intraspecific and interspecific communication may play key roles in forest insect communities' life histories and ecology, with closely and more distantly related species eavesdropping on each other’s signals.

## Data Availability

The data presented in this study are openly available at http://doi.org/10.6084/m9.figshare.16586261 (accessed on 1 October 2021).

## Supplementary Materials

The following are available online at https://www.mdpi.com/article/10.3390/insects12100899/s1, Table S1: Method for headspace sampling of *Plagionotus detritus*, Table S2: P-values of Kruskal–Wallis tests followed by Wilcoxon tests for the statistical analysis of data generated in Field test 1. 3-C6 indicates trap baits containing racemic 3-hydroxyhexan-2-one, (S)-2-C8 indicates trap baits containing enantiomeric (S)-2-hydroxyoctan-3-one, and (±)-3-C6+ (S)-2-C8 indicates the combination of the two, Figure S1: The parts of the VARb3 funnel trap. A: bait, B: upper funnel, C: funnel trap body, D: collection bucket.
