# Supplementary material for: Identification of Pheromone Components of Plagionotus detritus (Coleoptera: Cerambycidae), and Attraction of Conspecifics, Competitors, and Natural Enemies to the Pheromone Blend
Source: Insects. 2021 Oct 2;12(10):899. doi: 10.3390/insects12100899 (PMC8537793; doi:10.3390/insects12100899)
Supplement: Supplementary file 1 [file insects-12-00899-s001.zip › insects-1393615-SI.pdf]

Supplementary material

Identification of pheromone components of *Plagionotus detritus* (Coleoptera: Cerambycidae), and attraction of conspecifics, competitors, and natural enemies to the pheromone blend

Zoltán Imrei[1,*], Michael J. Domingue[2], Zsófia Lohonyai[1,3], Jardel A. Moreira[4], Éva Bálintné Csonka[1], József Fail[3], György Csóka[5], Lawrence M. Hanks[6], Miklós Tóth[1], Jocelyn G. Millar[7]

[1] Plant Protection Institute, Agricultural Research Centre, ELKH, H-1022 Budapest, Hungary; E-mail: ztimrei@gmail.com, lohonyai.zsofi@gmail.com, csonkaeva@yahoo.com, toth.miklos@atk.hu
[2] Department of Entomology, Kansas State University, 123 W. Waters Hall, Manhattan KS 66506, USA; michael.j.domingue@usda.gov
[3] Department of Entomology, Institute of Plant Protection, Hungarian University of Agriculture and Life Sciences, H-1118 Budapest, Hungary; fail.jozsef@uni-mate.hu
[4] Brainfarma Industria Química e Farmacêutica S.A, Barueri, São Paulo State, Brazil; jardel.moreira@yahoo.com.br
[5] Department of Forest Protection, Forest Research Institute, University of Sopron., H-3232 Mátrafüred, Hungary; csoka.gyorgy@erti.naik.hu
[6] Department of Entomology, University of Illinois at Urbana-Champaign, Urbana, IL 61801, USA; hanks@life.illinois.edu
[7] Department of Entomology, University of California, Riverside, CA 92521, USA; millar@ucr.edu

* Correspondence: E-mail: ztimrei@gmail.com, mobile: +36 70 571 8772 (Z.I.)

Table S1 Method for headspace sampling of *Plagionotus detritus*

Method/equipment	Description
Origin of beetles (site, coordinates[a])	Mátrafüred, Hungary, 47.8416N/19.9997E
Pre-collection conditions for the beetles	Outdoor temperature and light conditions, in ventilated transparent plastic boxes (56 × 28 × 28 cm), separated by sex, with oak shoots and apple slices
System type	Closed-loop stripping apparatus (CLSA)
Air pump (model, producer)	DC12/16NK, Erich Fürgut GmbH, Tannheim, Germany
Number of aeration containers	4
Containers (volume, producer)	0.5 l, hand-crafted glass, MOM, Budapest, Hungary
Airflow (l/min)	5.0
Collectors	1.5 mg charcoal, fixed with metal mesh in a glass tube (product: P/N 9 1006010, Brechbühler AG, Schlieren, Switzerland)
Aeration start	Between 8–10 AM
Duration	24 hr
Number of individuals per sex and aeration	3
Sexes separated	Yes
Blank control	Yes
Strips of fine metal mesh in the aeration containers	Yes
Light exposure during aeration	Sunlight
Extraction	100 μl dichloromethane
Cleaning of collectors	1) 4–5 ml methanol<br>2) 4–5 ml dichloromethane<br>3) 4–5 ml pentane
Rinsing glassware, metal mesh and tubing	1) methanol<br>2) dichloromethane<br>1) pentane
Storage of extracts	–54 °C, in PTFE seal screw cap vials (Sigma-Aldrich, Budapest, Hungary)

[a]Coordinates are given as decimal degrees (DD)

Table S2. P-values of Kruskal-Wallis tests followed by Wilcoxon tests for the statistical analysis of data generated in Field test 1. (±)-3-C6 indicates trap baits containing racemic 3-hydroxyhexan-2-one, (*S*)-2-C8 indicates trap baits containing enantiomeric (*S*)-2-hydroxyoctan-3-one, and (±)-3-C6+ (*S*)-2-C8 indicates the combination of the two.

species	sex	Kruskal -Wallis p-value	(±)-3-C6 vs unbaited	(*S*)-2-C8 vs unbaited	(*S*)-2-C8 vs (±)-3-C6	(±)-3-C6+ (*S*)-2-C8 vs unbaited	(±)-3-C6+ (*S*)-2-C8 vs (±)-3-C6	(±)-3-C6 +(*S*)-2-C8 vs (*S*)-2-C8
P. detritus	males	< 0.001	0.349	0.080	0.279	< 0.001	0.002	0.023
	females	< 0.001	0.164	0.164	0.974	< 0.001	< 0.001	< 0.001
	males & females	< 0.001	0.080	0.080	0.890	< 0.001	< 0.001	< 0.001
P. arcuatus	males	0.999						
	females	0.311						
	males & females	0.481						
X. antilope	males	0.053						
	females	< 0.001	1.00	< 0.001	0.001	0.025	0.029	0.177
	males & females	< 0.001	1.00	< 0.001	0.001	0.024	0.029	0.130
C. mutillarius	males	0.021	0.032	0.136	0.613	0.002	0.294	0.111
	females	0.022	0.002	0.009	0.588	0.009	0.693	0.828
	males & females	0.006	0.002	0.013	0.628	0.001	0.784	0.300

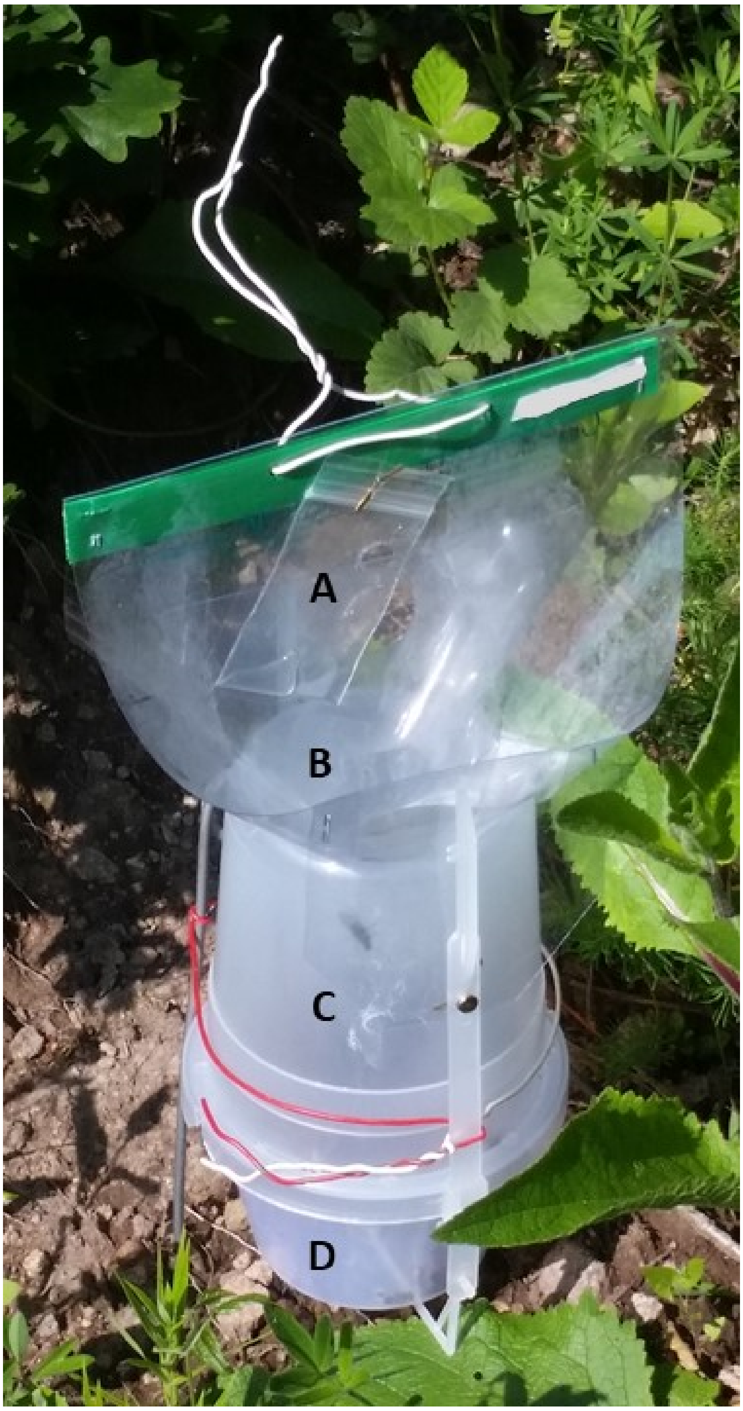

Figure S1. The parts of the VARb3 funnel trap. A: bait, B: upper funnel, C: funnel trap body, D: collection bucket.
